# Insights into the genetics of blood pressure in black South African individuals: the Birth to Twenty cohort

**DOI:** 10.1186/s12920-018-0321-6

**Published:** 2018-01-17

**Authors:** Liesl M. Hendry, Venesa Sahibdeen, Ananyo Choudhury, Shane A. Norris, Michèle Ramsay, Zané Lombard, of the AWI-Gen study and as members of the H3Africa Consortium

**Affiliations:** 1 School of Molecular & Cell Biology, Faculty of Science, University of the Witwatersrand, Johannesburg, South Africa; 2 Sydney Brenner Institute for Molecular Bioscience, Faculty of Health Sciences, University of the Witwatersrand, Johannesburg, South Africa; 3 Division of Human Genetics, School of Pathology, Faculty of Health Sciences, National Health Laboratory Service & University of the Witwatersrand, Johannesburg, South Africa; 4 MRC/Wits Developmental Pathways for Health Research Unit, Department of Paediatrics, School of Clinical Medicine, Faculty of Health Sciences, University of the Witwatersrand, Johannesburg, South Africa

**Keywords:** Birth to Twenty, black South Africans, blood pressure, genetics, Metabochip

## Abstract

**Background:**

Cardiovascular diseases (CVDs) are the leading cause of non-communicable disease deaths globally, with hypertension being a major risk factor contributing to CVDs. Blood pressure is a heritable trait, with relatively few genetic studies having been performed in Africans. This study aimed to identify genetic variants associated with variance in systolic (SBP) and diastolic (DBP) blood pressure in black South Africans.

**Methods:**

Genotyping was performed using the Metabochip in a subset of participants (mixed sex; median age 17.9) and their adult female caregivers (median age 41.0) from the Birth to Twenty cohort (*n* = 1947). Data were analysed as a merged dataset (all participants and caregivers together) in GEMMA (v0.94.1) using univariate linear mixed models, incorporating a centered relatedness matrix to account for the relatedness between individuals and with adjustments for age, sex, BMI and principal components of the genotype information.

**Results:**

Association analysis identified regions of interest in the *NOS1AP* (DBP: rs112468105 - *p* = 7.18 × 10^−5^ and SBP: rs4657181 - *p* = 4.04 × 10^−5^), *MYRF* (SBP: rs11230796 - *p* = 2.16 × 10^−7^, rs400075 - *p* = 2.88 × 10^−7^) and *POC1B* (SBP: rs770373 - *p* = 7.05 × 10^−5^, rs770374 - *p* = 9.05 × 10^−5^) genes and some intergenic regions (*DACH1|LOC440145* (DBP: rs17240498 - *p* = 4.91 × 10^−6^ and SBP: rs17240498 - *p* = 2.10 × 10^−5^) and *INTS10|LPL* (SBP: rs55830938 - *p* = 1.30 × 10^−5^, rs73599609 - *p* = 5.78 × 10^−5^, rs73667448 - *p* = 6.86 × 10^−5^)).

**Conclusions:**

The study provided further insight into the contribution of genetic variants to blood pressure in black South Africans. Future functional and replication studies in larger samples are required to confirm the role of the identified loci in blood pressure regulation and whether or not these variants are African-specific.

**Electronic supplementary material:**

The online version of this article (10.1186/s12920-018-0321-6) contains supplementary material, which is available to authorized users.

## Background

Cardiovascular diseases (CVDs) are the leading cause of non-communicable disease (NCD) deaths globally ahead of cancers, respiratory diseases and diabetes [1]. A major risk factor contributing to CVDs is hypertension, or raised blood pressure (BP). In 2014 the global prevalence of raised BP was approximately 22% in adults aged 18 years and older, with the highest prevalence reported in Africa at 30% for all adults combined [1]. BP and hypertension are heritable, multifactorial traits, with a significant genetic contribution (approximately 30–50% heritable [2]) in addition to various non-genetic risk factors. The genetic contribution is polygenic, with small contributions from risk alleles in multiple genes playing a role in the aetiology of the trait or disorder [3]. Numerous studies have already reported on genes and variants that have associations with hypertension, systolic blood pressure (SBP) and diastolic blood pressure (DBP). To date, however, most of the large-scale studies have been in individuals of European ancestry [4], with fewer studies conducted in individuals of Asian or African ancestry [5]. African-related research has also largely been carried out in African-Americans with studies conducted in cohorts from the African continent being limited.

The current study aims to elucidate the role of genetic polymorphisms in blood pressure variance in a black South African population.

## Methods

### Study participants

This study included all available DNA samples from African ancestry Birth to Twenty (Bt20) participants (mean age of 17.9 years with no minors at time of phenotype data collection) and their female caregivers (mean age of 41.9 years at time of phenotype data collection). The Bt20 cohort forms the basis of the largest longitudinal study on child and adolescent health and development in Africa. The cohort initially enrolled babies born as single births to women residing in Soweto, Johannesburg during a 7-week enrolment period between April and June 1990 and phenotype data has been collected at various time points since then [6].

Written assent was obtained from all participants at 13 years of age in conjunction with written consent from caregivers prior to blood sample collection. Written consent was obtained from participants when they were over 18 years of age. Ethical clearance was obtained from the University of the Witwatersrand Human Research Ethics Committee (Medical) for collection of DNA samples and phenotype data from this cohort (M010556). Further clearance was obtained for use of these samples to identify genetic risks associated with blood pressure (M1411116) in a black South African population. DNA is currently stored in the Division of Human Genetics at the National Health Laboratory Service (NHLS), Braamfontein, South Africa.

### Phenotype measurements

BP readings were taken and weight and height measured as described previously [7]. BP readings were taken with participants in a seated position. After five minutes of sitting in a resting position, three measurements were taken at intervals of two minutes. The first reading was discarded, in case of possible “white coat syndrome”, and an average of the second and third measurements was calculated and used in all analyses. Body mass index (BMI) was calculated as weight (kg) divided by height squared (m^2^). The phenotype data used in this study was from the year 13 and 17/18 data collection time points for the female caregivers and participants, respectively.

### Genotyping

DNA, extracted from blood using the salting out method [8], was normalized to 50 ng.ul^−1^ prior to genotyping at the UC Davis Genome Centre (California, USA) using the Metabochip (Illumina, San Diego, CA, USA). The Metabochip is a custom genotyping array that allows for the genotyping of almost 200,000 single nucleotide polymorphisms (SNPs) known to influence cardiometabolic traits [9]. The DNA samples were genotyped in two separate batches (participants and caregivers) and duplicate samples from each batch (nine in total) were sent with the unique samples to assess genotyping consistency. Genotypes were called using GenomeStudio Software for Illumina (v2011.1) and a custom DNAtech cluster file and final output was provided as final reports in the forward strand orientation.

### Data quality control

Pre-analysis quality control (QC) of the data was carried out separately for the two datasets using PLINK (v1.9) [10, 11], SMARTPCA (to run the principal component analysis (PCA) for identification of population outliers [12]) and Genesis (to visualize the PCA) [13]. Final report files were converted into binary PLINK format files. An initial SNP and sample removal step involved removing SNPs with complete missing genotype data and poorly genotyped samples (more than 20% missing genotype data). Further SNP QC involved removal of SNPs with high missingness rate (> 2%), low minor allele frequency (MAF) (< 0.01) and those failing Hardy-Weinberg equilibrium (HWE) (*p* < 1 × 10^−5^). Additional sample QC involved removal of samples with high missingness rate (> 2% for caregivers and >3% for participants), those with discordant sex, related samples (PI_HAT >0.1875), duplicates, samples with extreme heterozygosity (heterozygosity rate ± 3 standard deviations from the mean [14]) and population outliers (identified by manual inspection as those individuals falling significantly out of the main cluster in the PCA plots). One SNP (out of all those associated with DBP and SBP) was also disregarded due to poor clustering examined in the cluster plots generated in Evoker [15]. Checks were also carried out on the phenotype data and corrections were made where inconsistencies were found between the original questionnaires and captured data.

### Association analysis

Data were analysed as a merged dataset (all participants and caregivers together). Merging of the datasets in PLINK resulted in 1947 individuals and 125,906 SNPs remaining for analysis. Analysis was performed in GEMMA (v0.94.1) [16] using univariate linear mixed models and incorporating a centered relatedness matrix (calculated by GEMMA using the given genotypes) to account for the relatedness between individuals. BP was analysed as a continuous variable and included adjustments for age, sex and BMI. Principal components (PCs) were also included if necessary after examination of quantile-quantile (Q-Q) plots, which were constructed using R (v3.0.3) [17]. The number of PCs to include was determined by an improvement seen in the Q-Q plots after re-analysis with various numbers of PCs included: no PCs were deemed necessary to include for the analysis with DBP, while the first 10 PCs were included for the analysis with SBP. A Bonferroni array-wide significance threshold to correct for multiple testing was calculated as 0.05/number of independent markers: *p* < 6.7 × 10^−7^ (0.05/74475). The number of independent markers was calculated by performing linkage disequilibrium (LD) based SNP pruning where a window of 50 SNPs was considered at a time, LD between each pair of SNPs in the window was calculated and one of a pair of SNPs was removed if the LD was greater than 0.5. To address the possible introduction of type II errors through the application of this rigorous correction, we chose to also present results where a cut-off of *p* ≤ 1 × 10^−4^ was met, as results that may provide interesting biological leads.

## Results

This association analysis focused on 1947 African individuals (participants and their caregivers) from the Bt20 cohort. Descriptive statistics of the dataset are shown in Table 1.

**Table 1:** Descriptive statistics of the individuals remaining after QC in the merged dataset

		Mean (SD)	Median	Range
Merged (*n* = 1947)				
Females: 73.3%	Age (years)	30.1 (13.5)	30.0	17.3–84.0
Males: 26.6%	Weight (kg)	68.0 (16.7)	64.4	36.1–136.6
	Height (m)	1.6 (0.1)	1.6	1.2–1.9
	BMI (kg.m^−2^)	26.2 (7.1)	24.6	14.9–58.8
	SBP (mmHg)	117.8 (16.8)	116.0	77.0–206.5
	DBP (mmHg)	74.1 (11.1)	72.5	44.5–129.5

**Fig. 1:**
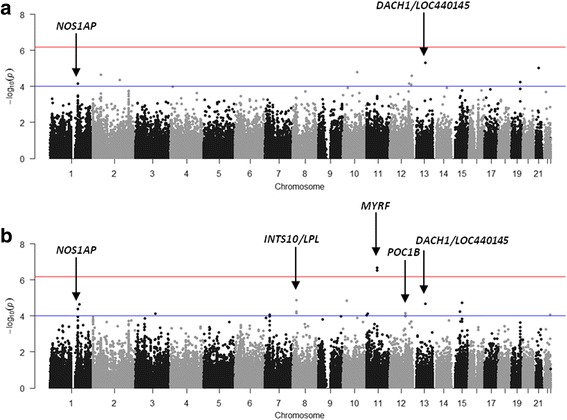
Manhattan plots drawn from the association results (with correction for covariates and PCs where necessary). Plots are shown for association with (**a**) DBP and (**b**) SBP. The upper horizontal line indicates the calculated array-wide significance cutoff (*p* = 6.7 × 10^−7^) while the lower horizontal line shows the cutoff of *p* = 1 × 10^−4^. Identified regions of interest for further investigation are indicated by arrows. *DACH1* – Dachshund Family Transcription Factor 1; *INTS10* – integrator complex subunit 10; *LPL* – lipoprotein lipase; *MYRF* – myelin regulatory factor; *NOS1AP* – nitric oxide synthase 1 (neuronal) adaptor protein; *POC1B* – POC1 centriolar protein

**Fig. 2:**
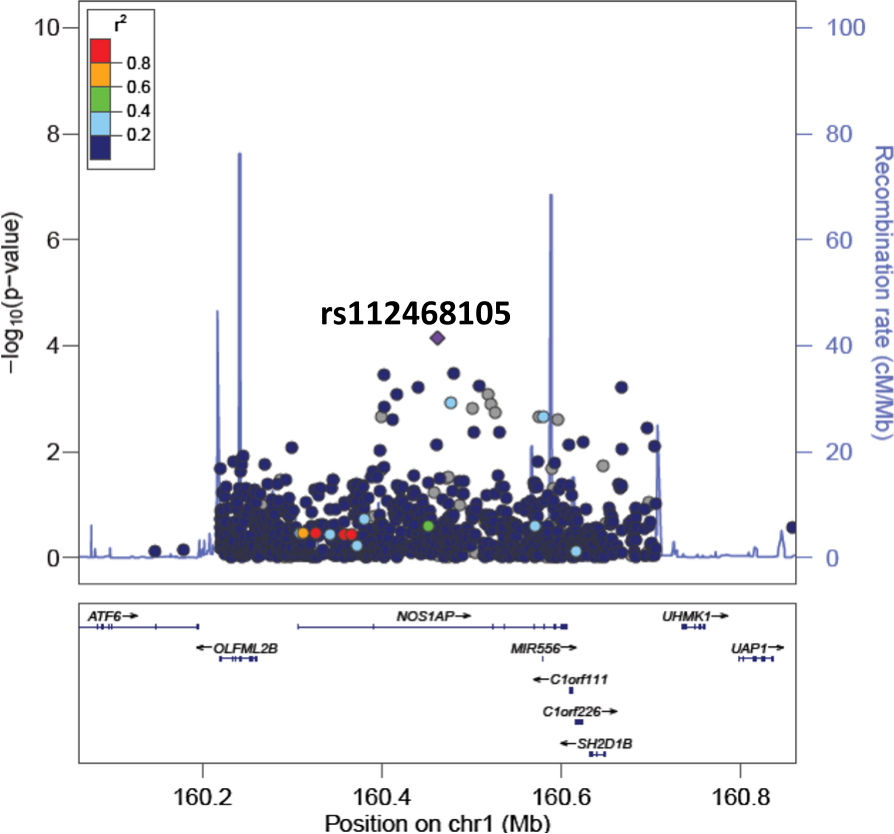
LocusZoom plot for the association of rs112468105 (in *NOS1AP*) with DBP against a YRI LD background. rs112468105 is represented by a purple diamond. SNPs around this index SNP are coloured according to the LD between each SNP and the index SNP. SNPs with missing LD information are shown in grey. rs112468105 is monomorphic in the CEU population

**Fig. 3:**
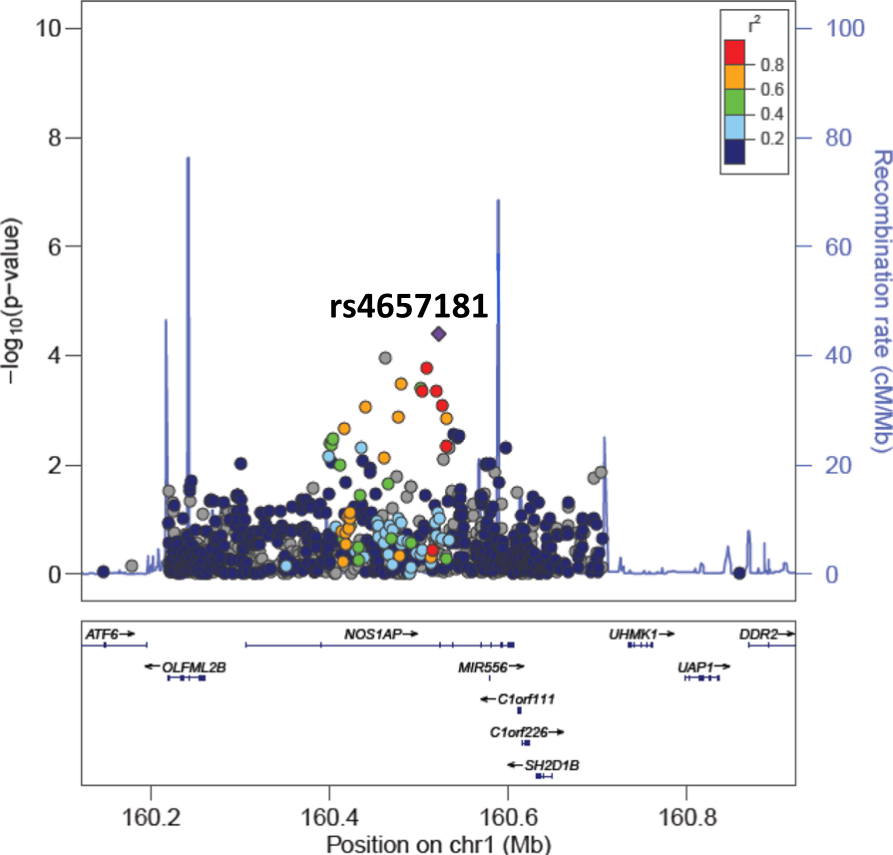
LocusZoom plot for the association of rs4657181 (in *NOS1AP*) with SBP against a CEU LD background. rs4657181 is represented by a purple diamond. SNPs around this index SNP are coloured according to the LD between each SNP and the index SNP. SNPs with missing LD information are shown in grey. The plot shows evidence of high LD in this region in the CEU population. rs4657181 had completely missing LD information when drawn for the YRI population

**Fig. 4:**
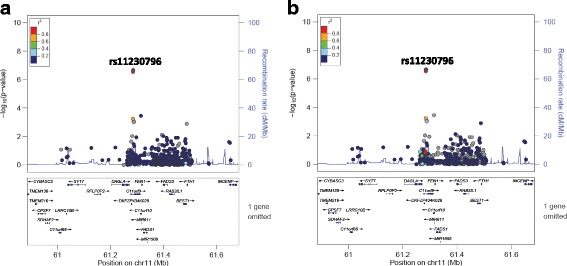
LocusZoom plots for the association of rs11230796 (in *MYRF*) with SBP against (**a**) YRI and (**b**) CEU LD backgrounds. rs11230796 is represented by a purple diamond. SNPs around this index SNP are coloured according to the LD between each SNP and the index SNP. SNPs with missing LD information are shown in grey. *MYRF* is referred to by an alternative name (*C11orf9*) in this plot. The plot shows evidence of high LD in both the YRI and CEU populations between rs11230796 and the other SNP (rs400075) that has a strong association with SBP

**Fig. 5:**
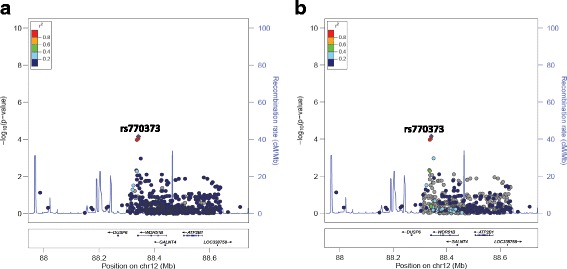
LocusZoom plots for the association of rs770373 (in *POC1B*) with SBP against (**a**) YRI and (**b**) CEU LD backgrounds. rs770373 is represented by a purple diamond. SNPs around this index SNP are coloured according to the LD between each SNP and the index SNP. SNPs with missing LD information are shown in grey. *POC1B* is referred to by an alternative name (*WDR51B*) in this plot. The plot shows evidence of high LD in both the YRI and CEU populations between rs770373 and the other SNP (rs770374) that has a strong association with SBP

**Fig. 6:**
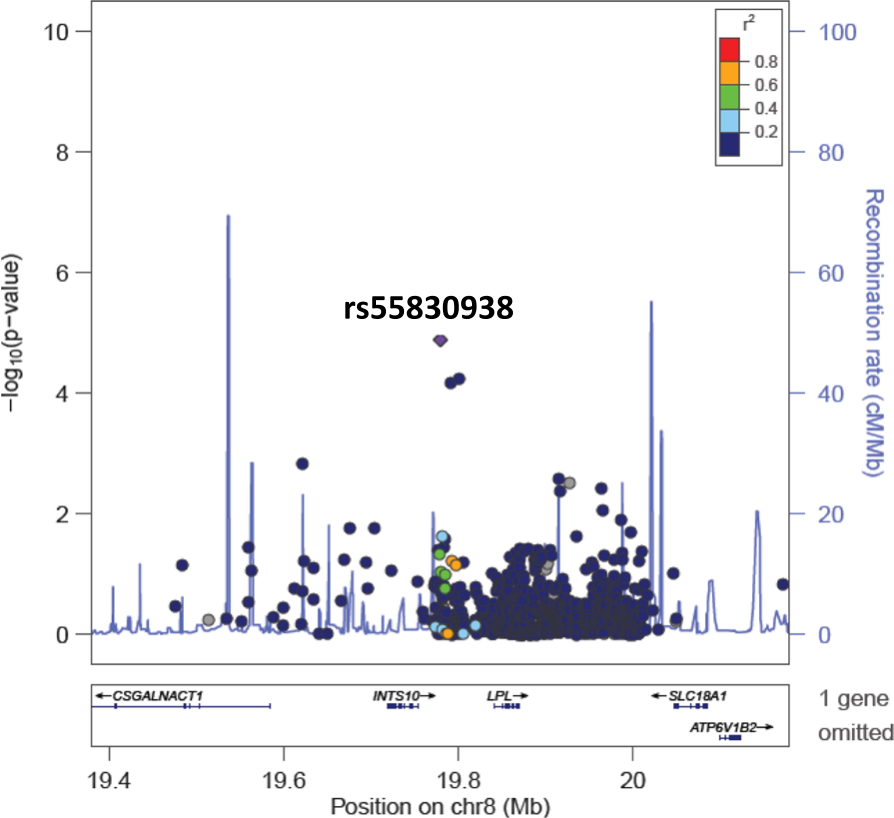
LocusZoom plot for the association of rs55830938 (intergenic to *INTS10* and *LPL*) with SBP against a YRI LD background. rs55830938 is represented by a purple diamond. SNPs around this index SNP are coloured according to the LD between each SNP and the index SNP. SNPs with missing LD information are shown in grey. rs55830938 is monomorphic in the CEU population

All SNPs associated with DBP or SBP at ≤1 × 10^−4^ are presented in Table 2. Interestingly, SNPs in introns in *NOS1AP* and intergenic to *DACH1|LOC440145* were found to be associated with both DBP and SBP. In addition, two SNPs intronic to *MYRF* and *POC1B* and three SNPs intergenic to *INTS10|LPL* associated with SBP. The only SNPs that reached array-wide significance are the two intronic SNPs in *MYRF* (rs11230796-G and rs400075-T) associated with SBP. Genome-wide visualisations of the associations, with these regions highlighted, are shown in the Manhattan plots in Fig. 1. Regional plots centered around the lead SNPs of *NOS1AP* (DBP and SBP), *MYRF* (SBP), *POC1B* (SBP) and the intergenic region of *INTS10|LPL* (SBP), and plotted against European (CEU) and Yoruban (YRI) LD backgrounds, are also shown (Figs. 2, 3, 4, 5 and 6).

**Table 2:** All SNPs associated with DBP or SBP at *p* ≤ 1 × 10^–4^

Phenotype	Chromosome	Gene/Region	SNP ID ^a^	Base pair position ^a^	Alleles		A1 Frequency ^c^			*P*-value ^d^	Beta ^e^
					A1 ^b^	A2	Bt20	YRI	CEU		
DBP	13	*DACH1 | LOC440145*	rs17240498	72,965,307	C	T	0.01	0.00	0.18	4.91 × 10^−6^	7.93
	21	*ADAMTS5 | C21orf94*	rs469709	28,801,007	A	G	0.05	0.02	0.13	9.21 × 10^−6^	3.50
	10	*LOC642666 | LOC727960*	rs12761063	82,533,425	T	C	0.11	0.12	0.10	1.58 × 10^−5^	2.47
	2	*PLEKHH2 | LOC728819*	rs13423605	43,886,460	C	T	0.13	0.18	0.04	2.25 × 10^−5^	2.29
	12	*SCARB1*	rs10846744	125,312,425	G	C	0.23	0.24	0.85	2.49 × 10^−5^	−1.79
	2	*ARL6IP6 | LOC391453*	rs2114653	153,648,587	G	A	0.16	0.14	0.36	4.28 × 10^−5^	−1.94
	19	*EML2 | GIPR*	rs4994276	46,164,172	T	C	0.15	0.20	0.19	5.87 × 10^−5^	−1.96
	12	*TRPV4*	rs16939725	110,250,587	G	A	0.14	0.14	0.02	7.08 × 10^−5^	−2.04
	1	*NOS1AP*	rs112468105	162,195,649	G	C	0.01	0.02	0.00	7.18 × 10^−5^	6.69
	12	*DNAH10*	rs6488908	124,377,814	G	A	0.13	0.14	0.04	7.94 × 10^−5^	−2.02
SBP	11	*MYRF*	rs11230796	61,529,267	G	T	0.06	0.06	0.22	**2.16 × 10** ^**−7**^	6.12
	11	*MYRF*	rs400075	61,528,814	T	C	0.06	0.06	0.22	**2.88 × 10** ^**−7**^	6.02
	8	*INTS10 | LPL*	rs55830938	19,735,188	G	T	0.03	0.03	0.00	1.30 × 10^−5^	7.49
	10	*LOC100128511 | C10orf114*	rs6482175	21,573,536	C	T	0.08	0.11	0.19	1.42 × 10^−5^	4.31
	13	*DACH1 | LOC440145*	rs17240498	72,965,307	C	T	0.01	0.00	0.18	2.10 × 10^−5^	11.68
	1	*FMO4 | BAT2D1*	rs10798391	171,389,938	T	G	0.02	0.01	0.19	2.19 × 10^−5^	8.16
	1	*NOS1AP*	rs4657181	162,255,385	T	A	0.05	0.02	0.56	4.04 × 10^−5^	−5.62
	15	*CYP19A1*	rs10459592	51,536,141	G	T	0.20	0.27	0.58	5.75 × 10^−5^	2.81
	8	*INTS10 | LPL*	rs73599609	19,756,974	C	G	0.05	0.05	0.00	5.78 × 10^−5^	5.14
	8	*INTS10 | LPL*	rs73667448	19,747,475	C	A	0.03	0.04	0.00	6.86 × 10^−5^	6.72
	12	*POC1B*	rs770373	89,818,289	T	C	0.18	0.33	0.45	7.05 × 10^−5^	−2.95
	11	*STK33*	rs1596888	8,455,344	T	G	0.43	0.48	0.72	7.51 × 10^−5^	2.20
	3	*C3orf17 | BOC*	rs1881941	112,852,476	A	T	0.12	0.72	0.93	7.66 × 10^−5^	3.39
	22	*FLJ46257 | FAM19A5*	rs6519991	48,725,535	A	G	0.17	0.16	0.10	8.31 × 10^−5^	−2.89
	7	*TAX1BP1*	rs6944913	27,826,523	G	A	0.02	<0.01	0.24	8.63 × 10^−5^	8.09
	11	*KCNQ1*	rs1557647	2,551,363	A	G	0.26	0.32	0.68	9.00 × 10^−5^	2.51
	12	*POC1B*	rs770374	89,818,022	T	G	0.23	0.37	0.56	9.05 × 10^−5^	−2.62

^a^All SNP IDs and base pair positions are reported using *GRCh37* (Genome Reference Consortium human genome Build 37)

^b^A1 corresponds to the minor allele in the dataset

^c^Frequencies of allele 1 are recorded for the dataset used in this study (Bt20) and for an African and European 1000 Genomes population - the Yoruba in Ibadan, Nigeria (YRI) and the Utah Residents with Northern and Western Ancestry (CEU)

^d^*p*-value adjusted for age, sex, BMI and principal components. *P*-values that pass the “array-wide” significance threshold (*p* < 6.7 × 10^−7^) are shown in bold

^e^Beta values are with respect to the minor allele in the sample. A positive beta indicates that the minor allele is associated with an increased blood pressure relative to the major allele, and *vice versa*

## Discussion

Much of the research focus in Africa has centred on infectious diseases, due to its high burden in this part of the world. NCDs are, however, gaining increasing interest and becoming as significant due to their increasing role in morbidity and mortality on the continent. This study aimed to investigate the genetics of BP in black South Africans and revealed several associations with DBP and SBP in black South Africans. The analysis pointed to regions of interest in the *NOS1AP* (DBP and SBP), *MYRF* (SBP) and *POC1B* (SBP) genes as well as two intergenic regions (*DACH1|LOC440145* and *INTS10|LPL*). Two SNPs in the *MYRF* gene met the array-wide significance threshold.

The gene with the most plausible functional link to BP regulation or hypertension risk in this study is the *NOS1AP* (nitric oxide synthase 1 (neuronal) adaptor protein) gene. In our study, two SNPs in this gene, rs112468105-G and rs4657181-T, associated with increased DBP and decreased SBP, respectively. Polymorphisms in this gene have previously been associated with other cardiovascular phenotypes, most notably QT interval length [18]. Interestingly, several genes in the chromosome 1q linkage region, in which *NOS1AP* falls, have previously been reported to be associated with hypertension [19] which motivates for this gene and regions on chromosome 1 to be investigated further for their role in BP/hypertension. The gene encodes an adaptor protein for neuronal nitric oxide synthase (nNOS), an enzyme involved in nitric oxide (NO) synthesis in the nervous tissue in the central and peripheral nervous systems. NO has several roles in the body, one of which is as a vasodilator and regulator of vascular tone and blood flow [20]. NO has also been implicated in BP regulation and impaired NO bioactivity has shown to be associated with hypertension, although the mechanism is unclear [21]. Research has also shown that NO synthesised in the central nervous system by nNOS is involved in the central regulation of blood pressure and inhibition of nNOS activity in the medulla and hypothalamus has been linked to systemic hypertension [22]. The exact role of the NOS1AP protein in the regulation of nNOS function in human disease is not clear, but a recent study showed that over-expression of NOS1AP increased nNOS activity [23].

Despite associations of the two SNPs in *MYRF (*myelin regulatory factor) (rs11230796-G and rs400075-T) with increased SBP at array-wide significance (*p* < 6.7 × 10^−7^), there is no clear functional link to BP. Polymorphisms in this gene have not shown any previous associations with BP or hypertension, but have been associated with fatty acid, phospholipid and blood metabolite levels [24–27].

There is some correlation of our findings with BP and other cardiometabolic-related trait associations found by studying European, Asian and African-American cohorts (Additional file 1: Table S1). Input of our top associated SNPs into the NHLBI GRASP catalog, v2.0.0.0 [28] and PhenoScanner [29] revealed one SNP (rs10798391) with a previous marginal association with SBP and DBP in European individuals [30]. rs10846744, associated with DBP in this study, showed previous associations with lipoprotein associated phospholipase A2 activity [31, 32] and rs11230796, associated with SBP at the array-wide significance level in this study, previously associated with serum linoleic acid and other polyunsaturated fatty acid levels in European individuals at a genome-wide significance level [33]. In addition, associations have been recorded between SNPs in *ARL6IP6* and SBP or DBP in African and Asian individuals [34, 35]; *MYRF* and DBP in Europeans [30]; *KCNQ1* and DBP, SBP or early onset hypertension in African, European or mixed populations [30, 36–38]; *NOS1AP, PLEKHH2* and *POC1B* and SBP or DBP in Europeans [30]; *SCARB1* and SBP in Europeans [30]; and *STK33* and *TAX1BP1* and hypertension in Europeans [39]. However, none of these associations were at a genome-wide significance level. Of importance to note is that our findings do not overlap with associations at a genome-wide significance level in large scale studies conducted in African Americans, including a recent study by Liang and colleagues [40].

It is interesting to note the marked difference in allele frequencies between this South African cohort and two other global populations (Table 2). Natural selection modulates the balance in allele frequencies across populations and variation in the frequencies of disease risk SNPs may help to explain racial disparities in disease risk [41]. It is surprising that some of the variants found to be associated with BP in this cohort are also observed at a high frequency in Europeans, yet have not been shown to be associated with BP risk in these populations. This could be explained by the differences in haplotype blocks seen in diverse populations, and the influence this may have on our ability to detect the true risk variant when using a genome-wide association study (GWAS) approach. It is now well established that association studies in Africans have the significant advantage that LD generally exists over a shorter genomic distance, potentially increasing the efficiency of the identification of causal variants [42].

The regional plots, generated in LocusZoom and centered around each of the lead SNPs in the identified regions of interest against YRI and CEU LD backgrounds, revealed high LD between several SNPs in *NOS1AP* in the CEU population (SBP) and high LD between the two associated SNPs in *MYRF* (SBP) and the two SNPs in *POC1B* (SBP) in both the YRI and CEU populations. The LD patterns in the South African population are likely to be similar and could explain the significant associations found in multiple SNPs within each region.

The Metabochip served as a good starting point in the investigation of BP genetics in black South Africans, despite the tool being developed from data on European populations. The Population Architecture using Genomics and Epidemiology (PAGE) study, whose main goal is to assess the generalizability of GWAS-identified variants across different populations, assessed the fine mapping capability of the Metabochip in African-Americans [43] and found it to be successful. Although it is known that African-Americans are genetically different to our African population, this was promising motivation for use in our black South African population. The Metabochip was developed in 2009 and several new BP/hypertension associated variants and regions have been identified since then, therefore possibly limiting the capacity to replicate in our population what has previously been found. In addition, as the Metabochip only contains variants known to be previously associated with cardiometabolic traits, the chances of identifying novel associations in Africans is reduced. A further limitation of the current study is the failure to account for the possible use of anti-hypertensive medication, specifically in the female caregivers.

## Conclusions

This study has provided interesting insight into the genetics of BP in black South Africans. Studies in larger samples could enable us to identify more associated variants that have modest to small effects. The functional significance of the associations identified is unclear, though some have plausible biological explanations for their role in regulating BP. Functional and replication studies in larger African studies, as are proposed within the H3Africa Consortium [44] and more specifically the AWI-Gen study [45], will no doubt provide more insight into the genetics of BP in African populations. In addition, generalisation analysis, which has proven to be informative in a recent study on BP genetics in Hispanic/Latino Americans (another understudied population) [46], can be performed in future analyses to investigate the genetic overlap of BP between populations.

## Additional file

Additional file 1: Table S1. Correlation of findings in our study with previous studies. (XLSX 13 kb)

## Abbreviations

*ARL6IP6*: ADP ribosylation factor like GTPase 6 interacting protein 6
AWI-Gen: African Wits-INDEPTH Partnership for the GENomic study of body composition and cardiometabolic risk
BMI: Body mass index
BP: Blood pressure
Bt20: Birth to Twenty
CEU: Utah Residents (CEPH) with Northern and Western European Ancestry
CVD: Cardiovascular disease
*DACH1*: Dachshund family transcription factor 1
DBP: Diastolic blood pressure
DNA: Deoxyribonucleic acid
GWAS: Genome-wide association study
H3Africa: Human Heredity and Health in Africa
HWE: Hardy-Weinberg equilibrium
*INTS10|LPL*: Integrator complex subunit 10/lipoprotein lipase
*KCNQ1*: Channel, voltage gated KQT-like subfamily Q, member 1
kg: Kilograms
LD: Linkage disequilibrium
m^2^: Metres squared
MAF: Minor allele frequency
*MYRF*: Myelin regulatory factor
NCD: Non-communicable disease
NHLS: National Health Laboratory Service
nNOS: Neuronal nitric oxide synthase
NO: Nitric oxide
*NOS1AP*: Nitric oxide synthase 1 (neuronal) adaptor protein
NRF: National Research Foundation
PAGE: Population Architecture using Genomics and Epidemiology
PCA: Principal component analysis
PCs: Principal components
*PLEKHH2*: Pleckstrin homology, MyTH4 and FERM domain containing H2
*POC1B*: POC1 centriolar protein B
QC: Quality control
Q-Q: Quantile-quantile
SBP: Systolic blood pressure
*SCARB1*: Scavenger receptor class B member 1
SNP: Single nucleotide polymorphisms
YRI: Yoruba in Ibadan, Nigeria
